# Arsenic Exposure Transforms Human Epithelial Stem/Progenitor Cells into a Cancer Stem-like Phenotype

**DOI:** 10.1289/ehp.0901059

**Published:** 2009-09-10

**Authors:** Erik J. Tokar, Bhalchandra A. Diwan, Michael P. Waalkes

**Affiliations:** 1 Inorganic Carcinogenesis Section, Laboratory of Comparative Carcinogenesis, National Cancer Institute at the National Institute of Environmental Health Sciences, National Institutes of Health, Department of Health and Human Services, Research Triangle Park, North Carolina, USA;; 2 Basic Research Program, SAIC-Frederick, Inc., National Cancer Institute-Frederick, Frederick, Maryland, USA

**Keywords:** arsenic, cancer stem cells, malignant transformation, prostate cancer, stem cells

## Abstract

**Background:**

Inorganic arsenic is a ubiquitous environmental carcinogen affecting millions of people worldwide. Evolving theory predicts that normal stem cells (NSCs) are transformed into cancer stem cells (CSCs) that then drive oncogenesis. In humans, arsenic is carcinogenic in the urogenital system (UGS), including the bladder and potentially the prostate, whereas in mice arsenic induces multiorgan UGS cancers, indicating that UGS NSCs may represent targets for carcinogenic initiation. However, proof of emergence of CSCs induced by arsenic in a stem cell population is not available.

**Methods:**

We continuously exposed the human prostate epithelial stem/progenitor cell line WPE-stem to an environmentally relevant level of arsenic (5 μM) *in vitro* and determined the acquired cancer phenotype.

**Results:**

WPE-stem cells rapidly acquired a malignant CSC-like phenotype by 18 weeks of exposure, becoming highly invasive, losing contact inhibition, and hypersecreting matrix metalloproteinase-9. When hetero-transplanted, these cells (designated As-CSC) formed highly pleomorphic, aggressive tumors with immature epithelial- and mesenchymal-like cells, suggesting a highly pluripotent cell of origin. Consistent with tumor-derived CSCs, As-CSCs formed abundant free-floating spheres enriched in CSC-like cells, as confirmed by molecular analysis and the fact that only these floating cells formed xenograft tumors. An early loss of NSC self-renewal gene expression (*p63*, *ABCG2*, *BMI-1*, *SHH*, *OCT-4*, *NOTCH-1*) during arsenite exposure was subsequently reversed as the tumor suppressor gene *PTEN* was progressively suppressed and the CSC-like phenotype acquired.

**Conclusions:**

Arsenite transforms prostate epithelial stem/progenitor cells into CSC-like cells, indicating that it can produce CSCs from a model NSC population.

Accumulating evidence indicates that cancer stem cells (CSCs) probably arise from normal stem cells (NSCs), or partially differentiated progenitor cells, and that CSCs are likely the driving force in tumor initiation, growth, and progression (Pardal et al. 2003; Reya et al. 2001). This developing theory is compelling because CSCs and NSCs share key characteristics, including the capacity for limitless self-renewal, which normally allows pluridirectional replenishment via differentiation but during oncogenesis contributes to heterogeneous aberrant cell overgrowth (Pardal et al. 2003). Moreover, NSCs are conditionally immortal and generally thought to be innately resistant to most toxic insults, likely facilitating survival selection and accumulation of molecular lesions required for acquired malignant phenotype, consistent with the multistep carcinogenesis model (Vogelstein and Kinzler 1993). Although CSCs share characteristics with tissue-concordant NSCs, they display dysregulated self-renewal (Bonnet and Dick 1997; Pardal et al. 2003; Ponti et al. 2005; Reya et al. 2001). Like NSCs, CSCs often form free-floating “spheres” of viable cells *in vitro* (Dontu et al. 2003; Ghods et al. 2007; Ponti et al. 2005; Tokar et al. 2005). Although assumed, proof of the direct emergence of epithelial CSCs from an NSC population is not available, and the identity of the cells that acquire the molecular lesions initiating chemical carcinogenesis remains undefined (Perez-Losada and Balmain 2003). Typically, to date, CSCs have been isolated from advanced tumors based on stem cell (SC)-like characteristics (Al-Hajj et al. 2003; Bonnet and Dick 1997; Ponti et al. 2005; Singh et al. 2004), rather than contemporaneously with carcinogenic initiation. Defining the role of CSCs in this primary triggering event is critical to a complete understanding of oncogenesis.

Inorganic arsenic is a widely distributed, naturally occurring environmental contaminant affecting tens of millions of people worldwide [International Agency for Research on Cancer (IARC) 2004]. Human or rodent arsenic exposure causes various urogenital system (UGS) cancers, including urinary bladder and kidney cancer (IARC 2004; Waalkes et al. 2007). Evidence in human populations in parts of Asia, the United States, and Australia exposed to arsenic suggests that the metalloid can target the prostate during arsenic-induced carcinogenesis (Benbrahim-Tallaa and Waalkes 2008); *in vitro*, inorganic arsenic causes malignant transformation of various cells, including human prostate cells (Achanzar et al. 2002; Benbrahim-Tallaa and Waalkes 2008). Arsenic also has transplacental carcinogenic activity in mice (Waalkes et al. 2007) and possibly in humans (Smith et al. 2006), with targets within the UGS (Waalkes et al. 2007). Because of relative fetal abundance and their role in organogenesis and differentiation, SCs appear to be key targets in transplacental carcinogenesis (Anderson et al. 2000) and are likely targets in transplacental arsenic carcinogenesis (Waalkes et al. 2008). Arsenic disrupts human and rodent skin SC dynamics *in vivo* and *in vitro*, eventually resulting in SC/CSC overabundance (Patterson and Rice 2007; Waalkes et al. 2008), an event probably significant to skin cancer development (Waalkes et al. 2008). Although arsenic is unequivocally carcinogenic, the mechanisms involved, including the precise target cells, remain enigmatic.

Leukemia SCs (LSCs) can be forced to arise from hematopoietic SCs (HSCs) after molecular manipulations of leukemogenesis genes (Krivtsov et al. 2006; Yilmaz et al. 2006; Zhang et al. 2006). However, it is unclear if human epithelial CSCs arise in a fashion similar to LSCs or if chemical carcinogens act through an attack on NSCs. Compared with other known carcinogens, a model prostate epithelial NSC population possesses a remarkable survival selection advantage toward arsenic over their differentiated isogenic counterparts (Tokar EJ, Waalkes MP, unpublished data), which would afford perpetuation but continued targeting during arsenic insult. Thus, we tested the hypothesis that arsenic targets prostate SCs as a central event in oncogenesis. For this, we selected the human SC/progenitor line WPE-stem derived from its parental, heterogeneous mature prostate line, RWPE-1, originally derived from normal prostate epithelium (Bello et al. 1997). WPE-stem cells have well-defined prostate SC/progenitor cell characteristics, including high p63 (tumor protein p63) and keratin 5/14 expression, and low androgen receptor, prostate-specific androgen, and K18 (keratin 18) expression (Tokar et al. 2005). WPE-stem cells form free-floating spheres (prostaspheres) in culture and have a high proliferation rate and relatively high colony-forming ability in agar (Tokar et al. 2005), typical NSC characteristics (Dontu et al. 2003; Ghods et al. 2007; Ponti et al. 2005). WPE-stem cells also highly express the prostate SC markers ABCG2 [ATP-binding cassette, subfamily G (WHITE), member 2] and BMI-1 [BMI1 polycomb ring finger oncogene] [see Supplemental Material, Figure 1, available online (doi:10.1289/ehp.0901059.S1 via http://dx.doi.org/)]. We used arsenic at a concentration that approximates levels in drinking water in areas where arsenicosis is common (Pi et al. 2002); and cells were exposed continuously, consistent with human exposure.

## Materials and Methods

### Cells, culture conditions, and arsenite exposure

RWPE-1 is a human papillomavirus (HPV)-18–immortalized, nontumorigenic, prostate epithelial cell line derived from normal adult human prostate (Bello et al. 1997). WPE-stem cells were isolated from RWPE-1 cells by single-cell dilution cloning, are nontumorigenic, and have extensive SC/progenitor cell characteristics [Tokar et al. 2005; see also Supplemental Material, Figure 1 and Table 1 (doi:10.1289/ehp.0901059.S1)], making them a good model for NSCs. Cells were maintained as previously described (Tokar et al. 2005), passaged once weekly (preconfluence), continuously exposed to a nontoxic arsenic concentration (5 μM, as sodium arsenite; Sigma, St. Louis, MO) for up to 18 weeks, and compared with unexposed passage-matched controls. Three separate flasks for each treatment were maintained.

### *In vitro* transformation

We assessed *in vitro* biomarkers of carcinogenic transformation every 3 weeks. Secreted matrix metalloproteinase-9 (MMP-9) activity was examined using conditioned medium as previously described (Tokar et al. 2005). Colony-forming efficiency (CFE) in soft agar, performed as previously described (Tokar et al. 2005), was used to assess acquired malignant phenotype. CFE also correlates with NSC or CSC phenotype (Stingl et al. 2006). We examined cellular invasiveness, another common characteristic of malignant cells, using a modified Boyden chamber invasion assay as described by Bello et al. (1997). When *in vitro* biomarkers of acquired cancer phenotype became sufficiently positive, *in vivo* xenograft studies were performed to establish malignant transformation.

### Free-floating sphere formation

Free-floating spheres of viable cells in culture are a characteristic of SCs and CSCs (Dontu et al. 2003; Ghods et al. 2007; Ponti et al. 2005; Tokar et al. 2005). To examine effects of arsenic on sphere formation, we plated cells (7.5 × 10^3^) in T-75 flasks; cells were then grown for 10 days and fed every 48 hr. Total spheres were then counted.

### Formation of branched ductal-like structures in Matrigel

Free-floating spheres were collected from flasks and dissociated into single-cell suspensions by pipette trituration then passage through a 40-μm cell strainer (BD Biosciences, San Jose, CA). Cells were suspended in 250 μL Matrigel (BD Biosciences) in 48-well plates and placed in incubators for 24 hr. Matrigel was then covered with 300 μL growth medium, which was changed every 48 hr. Images were taken 2 weeks later.

### Stem cell/differentiation biomarker expression during transformation

Total RNA was collected using TRIzol reagent (Invitrogen, Carlsbad, CA) and purified with RNeasy Mini Kit columns (Qiagen, Valencia, CA) according to the manufacturer’s protocol, and transcribed with MuLV (Moloney murine leukemia virus) reverse transcriptase and oligo-dT primers. Primers were designed with ABI Primer Express software (version 2.0; Applied Biosystems, Foster City, CA). We used SYBR Green Master Mix (ABgene, Rockford, IL) for real-time PCR (polymerase chain reaction) analysis. Cycle times were normalized with β-actin and glyceraldehyde-3-phosphate from the same sample and normalized to passage-matched controls. Genes examined included *p63* [tumor protein p63 (*TP63*)], *BMI*-*1*, *ABCG2*, *SHH* (sonic hedgehog), *OCT*-*4* [POU class 5 homeobox 1 (POU5F1)], *NOTCH*-*1* [Notch homolog 1, translocation- associated (*Drosophila*)], *K5* [keratin 5 (*KRT5*)], *K18*, and *PTEN* (phosphatase and tensin homolog). For gene and primer information, see Supplemental Material, Table 2 (doi:10.1289/ehp.0901059.S1). For Western blot analysis, protein extracts were collected using either NE-PER or M-PER extraction reagents (Pierce, Rockford, IL), separated by SDS-PAGE, transferred to polyvinyl difluoride membranes, and probed with anti-ΔNp63 (Santa Cruz Biotechnology, Santa Cruz, CA) or anti-cytokeratin-18 (Sigma) followed by horseradish peroxidase (HRP)-conjugated secondary antibodies (Santa Cruz Biotechnology). Membranes were stripped and reprobed with anti-β-actin antibody (Calbiochem, San Diego, CA) followed by peroxidase-conjugated anti-mouse (Santa Cruz Biotechnology). We used the ImageJ analysis program (version 1.24; National Institutes of Health 2007) for densitometric analysis.

### Immunocytochemical assessment of p63

We conducted immunocytochemistry for p63 protein as described previously (Tokar et al. 2005). Chamber slides (BD Biosciences) were coated with matrix (Tokar et al. 2005), and cells were plated and grown to subconfluence, washed with phosphate-buffered saline, and fixed with a 1:1 solution of methanol:acetone for 2 min. For staining, cells were blocked with normal horse serum (1 hr), incubated with primary antibody (ΔNp63, 1:100, 1 hr), rinsed, incubated with HRP-conjugated anti-mouse secondary antibody (1:500, 1 hr), and rinsed again. Staining was developed with ImmPACT DAB (diaminobenzidine) substrate (Vector Labs, Burlingame, CA), which produces a brown reaction product. Incubations were done at room temperature.

### Xenograft tumor formation

Once biomarkers of carcinogenic transformation suggested that arsenic-induced carcinogenic conversion had occurred, arsenic-treated and control cells were injected into male nude mice (NCr-*nu*; Charles River Laboratory, Wilmington, MA). Mice were housed at the NCI-Frederick animal facility (Frederick, MD), and animal care was provided in accordance with the Public Health Service policy on the care and use of animals (Institute of Laboratory Animal Resources 1996). For the first study, 1 × 10^6^ total cells (floating spheres and adherent cells) were collected and injected under the renal capsules of mice. In the second study, arsenite-treated floating cells were first separated from adherent cells and 1 × 10^6^ cells from each subgroup was injected subcutaneously (dorsal thoracic midline) into separate groups of mice. Animals were observed for tumor formation over a 6-month period.

### Statistical analysis

All data except tumor incidence represent mean and 95% confidence interval (CI). We used an unpaired Student’s *t*-test to compare arsenic-treated cells with untreated time-matched controls at individual time points. A Fisher’s exact test was used for tumor incidence data. In all cases, we consider a two-sided *p* < 0.05 significant. Sample sizes are given in figure legends.

## Results

### Arsenite-induced transformation of prostate SC/progenitor cells

**Figure 1:**
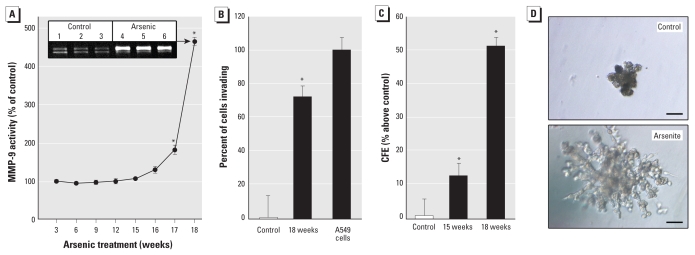
*In vitro* metrics of transformation in chronic arsenite-treated (5 μM) WPE-stem cells. (*A*) Secreted MMP-9 activity. Inset: representative zymogram (18 weeks). (*B*) Invasion; the A549 human lung carcinoma line was used as a positive control. (*C*) CFE; average numbers of colonies were 334 (control), 374 (15 weeks), and 504 (18 weeks). (*D*) Ductal/glandular-like structures produced by WPE-stem cells (top) or As-CSCs (bottom). Bars = 50 μm. See “Materials and Methods” for details. Numerical data represent mean and 95% CI (*n* = 3). *p < 0.05 compared with control..

MMP-9, an enzyme that when secreted digests extracellular matrix to aid in invasion and metastasis typical of cancer cells (Morelli et al. 2004), including those produced by arsenic (Achanzar et al. 2002), showed marked increases between 16 and 18 weeks of arsenic exposure in WPE-stem cells (Figure 1A). Marked increases in cellular invasion and CFE, which are additional characteristics of malignant cells, occurred concurrently (Figure 1B,C). In Matrigel, the WPE-stem cells formed ductal-like structures from single cells (Figure 1D), which is typical for NSCs from various tissues (Stingl et al. 2006), but the arsenite-treated structures grew much faster, were much more branched, and hence appeared much more “aggressive.” Together, these characteristics show that WPE-stem cells rapidly acquired a cancer phenotype with arsenite exposure.

**Figure 2:**
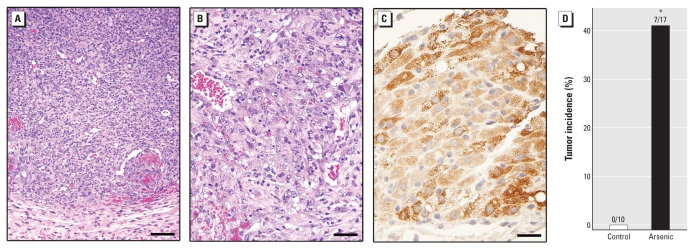
Xenograft tumors from arsenite-transformed WPE-stem cells. (*A, B*) Representative sections showing the highly undifferentiated, pleomorphic nature of tumors after As-CSC inoculation. Areas of red blood cells indicative of probable vasculature disruption, including potential point of access, are visible; bars = 100 μm in *A* and 50 μm in *B. B* is a representative tumor arising from As-CSC inoculation at a higher magnification than *A.* (*C*) Immunohistologic section showing K5, a prostate epithelial SC marker, in an As-CSC xenograft tumor, with strong, heterogeneous distribution in the epithelial and mesenchymal cells; bar = 25 μm. (*D*) Tumor incidence after inoculation under the renal capsule with control (*n* = 10) or arsenite-treated cells (18 weeks) (*n* = 17). *p < 0.05.

To establish malignant transformation, we inoculated cells under the renal capsules of mice. Arsenite-treated cells (As-CSCs) rapidly developed into highly pleomorphic tumors, with regional invasion and distant metastases that often dictated euthanasia in as little as 2–3 weeks. Tumors were highly undifferentiated, highly malignant, and composed of immature epithelial- and mesenchymal-like cells (Figure 2A,B). Strong heterogeneous staining for human K5 (Figure 2C), a marker of putative prostate epithelial SC/progenitor cells (Sawicki and Rothman 2002; Schalken and van Leenders 2003; van Leenders and Schalken 2001), supports a CSC-like nature and pluripotent cell of origination for these cancers. Tumors were very aggressive, showing invasion into renal parenchyma, abdominal muscle, adrenals, stomach, and small intestine, and very frequently metastasized to the lungs. During the 6-month observation period, no tumors occurred in mice inoculated with untreated cells, whereas tumor incidence was > 40% after inoculation with As-CSCs (Figure 2D).

### Arsenite causes overproduction of CSC-like cells

**Figure 3:**
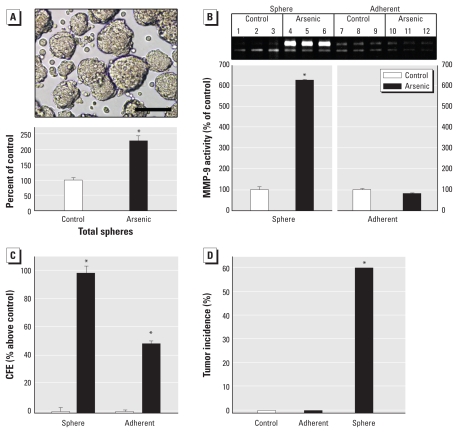
CSC-like characteristics in arsenite-treated and control cells. (*A*) Free-floating, viable spheres formed by arsenite-treated WPE-stem cells (top; bar = 100 μm) and sphere quantitation in control or arsenite-treated cells after malignant transformation (bottom). (*B*) Secreted MMP-9 activity from sphere and adherent cells from control and arsenite-treated cells after malignant transformation (bottom); the zymogram (top) shows MMP-9 activity. (*C*) Increased CFE in arsenite-transformed spheres after malignant transformation. Average numbers of colonies were 313 (control spheres), 637 (arsenite spheres), 326 (control adherent), and 479 (arsenite adherent). Data are percent increase compared with controls (mean and 95% CI; *n* = 3). (*D*) Tumor incidence in mice (*n* = 10/group) inoculated with control cells, arsenite-exposed adherent sphere cells, or arsenite-exposed sphere cells. *p < 0.05 compared with control.

A common characteristic of SCs in culture is the formation of floating “spheres” of viable cells (Dontu et al. 2003; Ghods et al. 2007; Ponti et al. 2005; Tokar et al. 2005). Malignantly transformed As-CSCs showed a 230% increase in free-floating spheres over control (Figure 3A). After separating floating and adherent cells, secreted MMP-9 activity from arsenite-exposed sphere cells was approximately 6 times that of either control (sphere or adherent) or arsenic-exposed adherent cells (Figure 3B). CFE of arsenite-exposed sphere cells was markedly elevated compared with adherent arsenite-exposed cells or either adherent or sphere control cells (Figure 3C). The cells driving tumor formation were present only in arsenite-exposed spheres (Figure 3D), because when sphere cells were first separated from adherent cells and then inoculated into mice, only arsenite-exposed sphere cells rapidly produced highly pleomorphic cancers (Figure 2D). Mice inoculated with arsenite-exposed adherent cells or pooled control cells showed no tumor formation when euthanized 6 months after inoculation.

### Self-renewal gene suppression and then reactivation during acquired CSC-like phenotype

**Figure 4:**
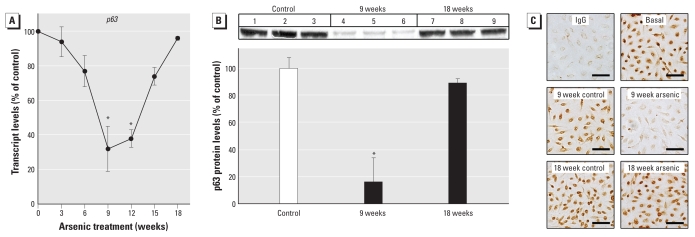
Expression of *p63*, an NSC gene associated with maintenance and self-renewal, during arsenite-induced malignant transformation of WPE-stem cells. *p63* transcript (*A*) and protein (*B*) levels decreased at 9 weeks but increased again to control levels by point of malignant transformation (18 weeks). Data represent mean and 95% CI (*n* = 3–6). (*C*) Immunocytochemical images for p63 consistent with Western blots show that WPE-stem cells remained a homogeneous population during transformation; bars = 50 μm. *p < 0.05 compared with time-matched controls (*A*) or baseline levels (*B*).

**Figure 5:**
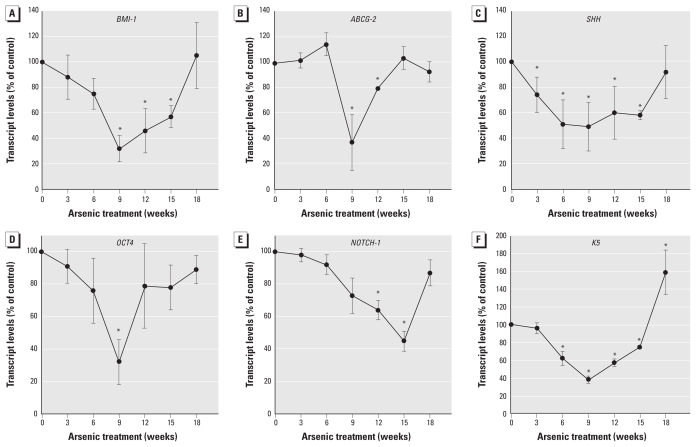
Expression of NSC genes associated with maintenance, self-renewal, and differentiation during arsenite-induced malignant transformation of WPE-stem cells. Transcript levels for *BMI-1* (*A*), *ABCG2* (*B*), *SHH* (*C*), *OCT-4* (*D*), *NOTCH-1* (*E*), and *K5* (*F*) all show the same loss then reactivation of expression as that seen with *p63*, indicating that a common pattern of alterations in an NSC-like transcription program involving self-renewal occurs during arsenite-induced progression of NSCs to CSCs. Data represent mean and 95% CI (*n* = 3–6). *p < 0.05 compared with time-matched controls.

**Figure 6:**
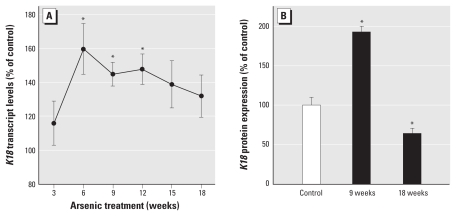
K18 expression during arsenic-induced malignant transformation of WPE-stem cells. (*A*) *K18* transcript levels during chronic arsenic exposure show early, marked increases followed by a decrease back toward control levels by the time of malignant transformation (18 weeks; *n* = 6). (*B*) Quantitative analysis of K18 protein levels during malignant transformation (*n* = 3) shows that the trend is consistent with transcript levels and is opposite that seen for SC markers (Figures 4 and 5). Data represent mean and 95% CI. *p < 0.05 compared with time-matched controls (*A*) or baseline levels (*B*).

We examined expression of several NSC self-renewal genes during arsenite-induced CSC phenotype acquisition. A remarkably consistent temporal pattern emerged in which NSC self-renewal gene expression was first lost and then reactivated with arsenite-induced acquired CSC-like phenotype (Figures 4 and 5). As arsenite transformed NSCs into As-CSCs, the expression of self-renewal– associated genes *p63* (Figure 4), *BMI-1*, *ABCG2*, *SHH*, *OCT-4*, and *NOTCH-1* (Figure 5A–E) was first markedly suppressed (~ weeks 1–9) then reactivated (~ weeks 9–18). Additionally, *K5*, a marker of undifferentiated prostate SC/progenitor cells, showed the same pattern (Figure 5F). Conversely, *K18*, a marker of differentiated cells, showed the opposite trend of NSC self-renewal genes, first increasing and then returning to basal levels with transformation (Figure 6). This indicates that SC self-renewal was initially lost during early transformation, potentially due to aberrant differentiation, and then regained with malignant transformation, consistent with distorted self-renewal common to CSCs (Pardal et al. 2003; Reya et al. 2001). This is consistent with CSC overproduction induced by arsenite and the aggressive, highly pluripotent tumors formed by As-CSC cell inoculation. WPE-stem cells normally show homogeneous expression of prostate SC marker p63 (Tokar et al. 2005), and immunocytochemical assessment showed that, although exposure-duration–related expression changes occurred, expression remained homogeneously distributed throughout arsenite-induced transformation (Figure 4C), indicating that subpopulation selection did not occur.

### Arsenite suppresses PTEN

**Figure 7:**
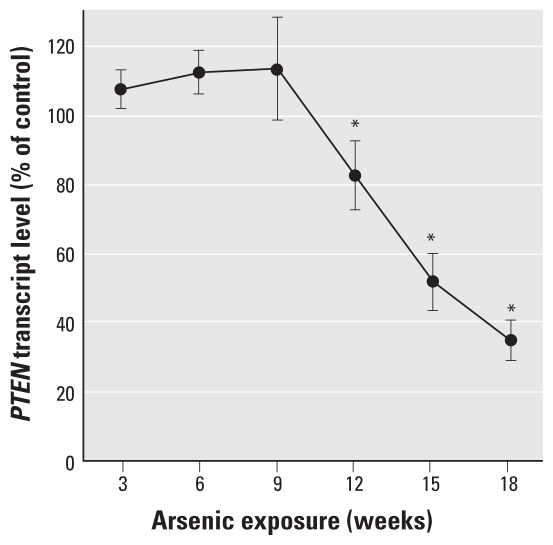
*PTEN* expression during arsenite-induced malignant transformation of WPE-stem cells. *PTEN* transcript levels show a progressive and marked decrease starting at 9 weeks of arsenite treatment. Data represent mean and 95% CI (*n* = 6). *p < 0.05 compared with time-matched controls.

*PTEN*, a tumor suppressor gene that modulates cell signaling pathways, has critical roles in SC differentiation and is often inactivated in malignancies (Di Cristofano and Pandolfi 2000). Starting at 9 weeks of arsenite exposure, a rapid, progressive decrease in *PTEN* expression occurred (Figure 7). This decrease coincided with the reactivation of the SC self-renewal genes, indicating that these events are likely linked.

## Discussion

Human arsenic exposure is linked to various UGS tumors, potentially including prostate cancers (Benbrahim-Tallaa and Waalkes 2008; IARC 2004), and emerging data indicate that arsenic may have human transplacental carcinogenic activity (Smith et al. 2006). Similarly, prenatal arsenic exposure in mice induces or initiates neoplastic lesions throughout the UGS in adulthood (Waalkes et al. 2007, 2008), and because SCs may be an important target in transplacental carcinogenesis (Anderson et al. 2000), this suggests that UGS NSCs are key targets of arsenic. Using a model prostate epithelial NSC population, we found that the SC phenotype appears to have a strong survival selection advantage toward arsenite (Tokar EJ, Waalkes MP, unpublished data), likely allowing for survival in the face of continued carcinogenic insult. Based on these observations, in the present study an established human prostate SC/progenitor cell line (Tokar et al. 2005) was chronically exposed to a nontoxic, environmentally relevant concentration of arsenite (Pi et al. 2002) and rapidly (18 weeks) produced malignant transformant cells that showed multiple characteristics of CSCs, proved highly aggressive, and produced xenograft tumors indicative of a remarkably pluripotent cell of origin. This is compelling evidence that the metalloid can act directly upon a human SC/progenitor population to initiate formation of CSC-like cells and provides unique insights into the very earliest period of arsenic carcinogenesis. In contrast, the same level of arsenite will induce transformation in its isogenic, mature heterogeneous parental RWPE-1 cell line, but this takes 30 weeks, and the cells form much more differentiated tumors of epithelial origin with mature prostate qualities [Achanzar et al. 2002; see Supplemental Material, Table 1 (doi:10.1289/ehp.0901059.S1)]. As further examples, *in vitro* arsenic-transformed RWPE-1 cells show relatively modest increases in MMP-9 secretion (Achanzar et al. 2002) and relatively low invasiveness and colony formation (Tokar EJ, Waalkes MP, unpublished data) compared with As-CSC cells. Thus, the WPE-stem cells transform much faster and show a much more aggressive and totipotent phenotype (present study) compared with mature heterogeneous isogenic parental transformed cells (Achanzar et al. 2002). In accord with the CSC theory (Gisselsson 2007; Pardal et al. 2003; Reya et al. 2001) and the multistep carcinogenesis theory (Vogelstein and Kinzler 1993), the stage of differentiation determines not only the number of genetic alterations necessary for carcinogenic transformation but also dictates the aggressiveness of resulting tumors (Gisselsson 2007). NSCs, with their permissive state of differentiation, would need much fewer molecular transformational events, whereas mature cell populations would require more numerous alterations (Gisselsson 2007). The much more rapid transformation and resultant aggressive, pluripotent phenotype seen in arsenite-transformed As-CSC cells compared with the transformed mature, parental cells (Achanzar et al. 2002) is consistent with this “permissive plasticity” concept of SCs in chemical carcinogenesis theory (Gisselsson 2007).

The arsenic biomethylation capacity of WPE-stem cells is unknown. However, the parental RWPE-1 cells only very poorly methylate arsenic (Benbrahim-Tallaa et al. 2005). Whether the transformative agent of WPE-stem was inorganic arsenic, a methylated metabolite, or some combination remains to be determined.

CSCs isolated from advanced cancers typically reinitiate their tumor of origin (Al-Hajj et al. 2003; Singh et al. 2004). The remarkably low level of differentiation of As-CSC–produced xenograft tumors is perhaps a result of being transformed in the absence of surrounding mature cell signals directing them to a particular cancer based on tissue of origin. Such a minimally differentiated status is rare in adulthood tumors but common in some childhood tumors (e.g., nephroblastomas, teratomas) that are derived from immature embryonal-like cells (Gisselsson 2007). This primordial epithelial/mesenchymal histopathology, coupled with strong heterogeneous staining for epithelial SC markers (K5; Schalken and van Leenders 2003), suggests that the As-CSC hetero-transplantation tumors developed from highly pluripotent cells, allowing divergent differentiation toward both epithelial and mesenchymal components. The rapid progression and high aggressiveness suggest that arsenite quickly triggered acquisition of a malignant CSC-like phenotype consistent with a loss of self-renewal and differentiation control. Similarly, pleomorphic uterine carcinosarcomas, although not associated with arsenic exposure in humans, were once thought to be “collision” tumors but are now thought to derive from a single SC population that has acquired the ability to undergo aberrant, divergent differentiation, producing both mesenchymal and epithelial cancerous components (McCluggage 2002). Our data indicate that arsenite has instilled a similar potential into As-CSC cells. Furthermore, very recently we found that when injected intravenously into mice, As-CSC cells formed highly pleomorphic malignancies in lung, liver, and skin (Tokar EJ, Waalkes MP, unpublished data). This provides significant support to the concept that arsenic has transformed human NSC/progenitor cells into CSC-like cells and adds credence to this as a plausible component of the mechanism of arsenic carcinogenesis.

Many cultured NSC or CSC lines form free-floating spherical clusters of viable cells containing a preponderance of the NSCs or CSCs (Dontu et al. 2003; Ghods et al. 2007; Ponti et al. 2005; Tokar et al. 2005). Only sphere cells from arsenite-transformed WPE-stem cells formed xenograft tumors. This strongly supports our contention that arsenite has triggered CSC-like formation from phenotypically normal SCs because tumor-forming CSCs derived from advanced cancers typically reside in these spheres (Ghods et al. 2007). Furthermore, arsenite produced an overabundance of CSC-like cells, primarily in the spheres, potentially accounting for the rapid formation, totipotent nature, and remarkable aggressiveness of the resulting xenograft tumors. *In vivo* arsenic alters skin SC population dynamics, eventually enhancing skin carcinogenesis coinciding with increased CSCs in epidermal carcinomas (Waalkes et al. 2008). Thus, arsenic appears to target SC populations both *in vitro* (present study; Patterson and Rice 2007) and *in vivo* (Waalkes et al. 2008), distorting SC dynamics and precipitating CSC overproduction. Whether other carcinogens act in a similar fashion is an important question that requires further investigation.

NSCs and CSCs share fundamental properties, including self-renewal capacity, which is typically dysregulated in oncogenesis (Pardal et al. 2003; Reya et al. 2001). In this regard, a remarkably consistent “U-shaped” temporal response occurred with several SC self-renewal genes (*p63*, *BMI-1*, *ABCG2*, *SHH*, *OCT-4*, and *NOTCH-1*) during arsenic-induced malignant transformation, with an initial suppression followed by reactivation. p63 is essential for SC self-renewal and proliferation within the prostate and other tissues (Senoo et al. 2007; Signoretti et al. 2005). BMI-1 is required for self-renewal of both NSCs and CSCs in the prostate (Lessard and Sauvageau 2003; Pardal et al. 2003). In several tissues SHH promotes SC self-renewal, whereas aberrant activation is implicated in carcinogenesis (Reya et al. 2001; Ruiz i Altaba et al. 2002). ABCG2 plays a clear role in maintaining SC populations and is a prostate NSC or CSC marker (Huss et al. 2005; Zhou et al. 2001). *OCT-4* is a pluripotency gene required for NSC self-renewal and maintenance that is often activated in carcinogenesis (Hochedlinger et al. 2005). The Notch signaling pathway regulates NSC self-renewal and differentiation and is frequently dysregulated in oncogenesis (Artavanis-Tsakonas et al. 1999; Pardal et al. 2003). In the prostate, Notch-1 is a marker for SC/progenitor cells, is required for normal development, and has been shown to function as both an oncogene and tumor suppressor during prostate carcinogenesis (Leong and Gao 2008). Additionally, for keratins, K5 is a marker for undifferentiated basal SCs, whereas K18 is a marker of differentiated cells, and K5 expression increases during carcinogenesis (Sawicki and Rothman 2002; Schalken and van Leenders 2003). The remarkably consistent trend in these critical self-renewal/differentiation genes during WPE-stem transformation suggests that arsenite instilled aberrant self-renewal and differentiation capabilities as these cells acquired a malignant CSC phenotype. Intriguingly, this trend is consistent with that seen when LSCs are formed by introduction of the mixed lineage leukemia (*MLL-AF9*) gene into hematopoietic committed progenitor cells (Krivtsov et al. 2006). A group of self-renewal genes highly expressed in HSCs is lost in committed progenitors but reactivated in LSCs initiated by introduction of the MLL-AF9 fusion protein (Krivtsov et al. 2006). Likewise, reactivation of an SC-like genetic program in adult hematopoietic cells can introduce aberrant self-renewal characteristics of CSCs (Wong et al. 2008). Thus, distortion of an NSC self-renewal-associated signature drives these hematopoietic cells toward LSC phenotype (Krivtsov et al. 2006; Wong et al. 2008) and clearly shows that this trend is not arsenic specific, but potentially common during the process of CSC formation. Together with prior work (Krivtsov et al. 2006; Wong et al. 2008), the present data suggest that sequential distortion of self-renewal–associated genes is critical to CSC formation.

During arsenite-induced transformation and formation of As-CSCs, the dramatic decrease in *PTEN* expression coincided with the reactivation of self-renewal genes and acquisition of malignant phenotype. Poor prostatic *PTEN* expression increases SC-like cells and causes cancer initiation (Signoretti and Loda 2007) and in prostate cancer cells increases sphere-forming ability and xenograft sphere cell tumorigenicity (Dubrovska et al. 2009). Furthermore, loss of *PTEN* enhances self-renewal capacity of SCs without dramatically altering pluripotency (Groszer et al. 2006). *PTEN* expression maintains HSCs in a quiescent state, and its deletion leads to HSC depletion and leukemia formation enriched for LSCs (Yilmaz et al. 2006; Zhang et al. 2006). The loss of *PTEN* provides a plausible mechanism through which As-CSC cells reactivate self-renewal, although dysregulated, and yet maintain and potentially distort multipotential differentiation capacity.

## Conclusion

Our data support the hypothesis that invokes emergence of CSCs from NSCs as a primal initiating event in oncogenesis (Bapat 2007; Pardal et al. 2003; Reya et al. 2001), at least for arsenic. Indeed, arsenite directly targeted the human WPE-stem line, transforming them into a highly aggressive CSC-like phenotype. Once transformed, these cells possess multiple CSC-like characteristics, including enhanced self-renewal capacity, competency to proliferate, clonogenicity, and, perhaps most important, the ability to initiate highly aggressive, immature, pluripotent tumors *in vivo*. The truly stunning capacity to rapidly produce highly pleomorphic tumors in xenograft studies and the fact that only cells from the arsenite-treated spheres formed tumors, together with strong evidence that CSCs reside in similar spheres derived from advanced cancers (Ghods et al. 2007), support a direct acquisition of malignant CSC-like phenotype from NSC/progenitor cells triggered by arsenic. Additionally, our data fortify the emerging concept that the loss and subsequent reactivation of SC self-renewal are critical during malignant transformation of SCs, as appears to be the case with leukemias (Bapat 2007; Krivtsov et al. 2006; Rossi et al. 2008; Wong et al. 2008).

Overall, this study expands our understanding of the carcinogenic potential of arsenic, a common environmental contaminant (IARC 2004), by indicating that it can directly target SCs for carcinogenic transformation. Millions of people are exposed, likely throughout their entire lifetime, to unhealthy levels of arsenic in drinking water (IARC 2004). Arsenic is clearly a transplacental carcinogen in rodents (Waalkes et al. 2007) and probably in humans (Smith et al. 2006), and SCs are likely key targets in *in utero* life-stage susceptibility to chemical carcinogenesis (Anderson et al. 2000). Therefore, the ability to malignantly transform NSCs could be of major mechanistic significance in arsenic-exposed human populations.
